# The Book World of Medicine and Science

**Published:** 1896-02-29

**Authors:** 


### THE BOOK WORLD OF MEDICINE AND SCIENCE.

Modern Medicine and Homœopathy. By John B. Roberts, M.D. (The Edwards Docker Company, Philadelphia. 1895.) Price 4s.

As stated on the title page, this book deals first with the points of similarity between orthodox physicians and homœopathists, and then considers the present attitude of modern medicine and those who practise it towards homœopathy. We cannot say that the result is satisfactory. The author proves up to the hilt that a great deal of the homœopathy which we hear of is such only in name, and that those who pretend to practise it are really engaged in the ordinary practice of medicine on ordinary principles. The great body of homœopathic practitioners, it says, "use any drug administered in any way that seems to them likely to be beneficial. They are, however, called homœopaths because they have a belief in the partial value of a law of similars." Quotations are given from homœopathic journals to the same effect as follows: "The practitioners of homœopathy forty years ago who are now living can scarcely recognise the merchantable article called homœopathy at the present day." The *Homœopathic News* for March, 1892, says editorially, "We venture to assert that had not our school drifted away from the practice of forty years ago, it would have been dead and buried long since." "We have drifted away from the practice of giving a pellet of the two-hundredth or higher and waiting thirty or sixty days for its curative effects; from the prescribing of a high dilution by smelling the dry pellets, those same pellets 'grafted' by shaking a thousand pure pellets with one medicated by the ten-thousandth." "We have drifted away from the carrying a pocket repertory to the bedside of the patient, and recording the symptoms in columns, and a weary search in said repertory until a mechanical similium was found." All this may be very true, as, in fact, we believe it is, and Dr. Bell, the president of the International Hahnemannian Association, is probably quite correct in his lament that three hundred would be a generous estimate for the total number in the whole world of those "who may be called true Hahnemannians or who are becoming such." Nevertheless the moral which this book is intended to teach does not seem any the more acceptable. Ordinary practitioners are asked to hold out the hand of fellowship to the men who professedly are practising a sham system of homœopathy, but they may fairly ask in return that these men should first give up a name which, so far as it does not represent their ordinary practice, is but a pretence. It is suggested that if the societies composed of non-sectarian physicians would revise their by-laws, so that physicians now called homœopathists might be eligible for membership, the next move should be for the homœopathic medical societies to drop the sectarian name. But surely this is putting the cart before the horse.

The Year-book of Treatment for 1896. (Cassell and Co., London. 1896.)

This well-known annual, although this year shorter by a few pages than last, fully maintains its reputation as a reliable and convenient review of medical literature so far as it concerns itself with treatment. We notice that this edition contains a supplement giving a short account of the new drugs, instruments, and dietetic products introduced by various well-known firms. The list of new books which is added will probably be useful to those who make the "Year-book" an annual addition to their shelves, although it must be confessed that reference to no single volume gives a bibliography which can be of much service in regard to any given subject. The work, however, is an admirable summary, is up to date, is well indexed, and fills a place of great utility as a ready book of reference, not only giving in brief the progress of the year, but showing in each case where to apply for more complete information.

Kelly's London Medical Directory, 1896. (Kelly and Company, High Holborn.)

We have received a volume of "Kelly's Medical Directory" for the present year. It appears in every respect to have maintained its character for usefulness and accuracy, and should find a place in the library of all families residing in the metropolis or suburbs.

Science Progress.

The March number of *Science Progress* will contain an interesting article on Ludwig—Modern Physiology—by Professor Burdon Sanderson, and one on "Recent Advances in Vegetable Cytology" by Professor J. Bretland Farmer.

